# The Potential of *Spirulina platensis* to Ameliorate the Adverse Effects of Highly Active Antiretroviral Therapy (HAART)

**DOI:** 10.3390/nu14153076

**Published:** 2022-07-27

**Authors:** Thabani Sibiya, Terisha Ghazi, Anil Chuturgoon

**Affiliations:** Discipline of Medical Biochemistry and Chemical Pathology, School of Laboratory Medicine and Medical Sciences, College of Health Sciences, University of KwaZulu-Natal, Howard College Campus, Durban 4013, South Africa; thabani585@gmail.com (T.S.); terishaghazi@gmail.com (T.G.)

**Keywords:** HAART/ARVs, *Spirulina platensis*, oxidative stress, HIV, antioxidant, inflammation, HAART toxicity, MetS

## Abstract

The human immunodeficiency virus (HIV) is one of the most prevalent diseases globally. It is estimated that 37.7 million people are infected with HIV globally, and 8.2 million persons are infected with the virus in South Africa. The highly active antiretroviral therapy (HAART) involves combining various types of antiretroviral drugs that are dependent on the infected person’s viral load. HAART helps regulate the viral load and prevents its associated symptoms from progressing into acquired immune deficiency syndrome (AIDS). Despite its success in prolonging HIV-infected patients’ lifespans, the use of HAART promotes metabolic syndrome (MetS) through an inflammatory pathway, excess production of reactive oxygen species (ROS), and mitochondrial dysfunction. Interestingly, *Spirulina platensis* (SP), a blue-green microalgae commonly used as a traditional food by Mexican and African people, has been demonstrated to mitigate MetS by regulating oxidative and inflammatory pathways. SP is also a potent antioxidant that has been shown to exhibit immunological, anticancer, anti-inflammatory, anti-aging, antidiabetic, antibacterial, and antiviral properties. This review is aimed at highlighting the biochemical mechanism of SP with a focus on studies linking SP to the inhibition of HIV, inflammation, and oxidative stress. Further, we propose SP as a potential supplement for HIV-infected persons on lifelong HAART.

## 1. Introduction

The human immunodeficiency virus (HIV) has continued to be a global public concern due to its widespread infection rate and alarming mortality rate [1]. The Joint United Nations Programme on HIV/AIDS (UNAIDS), in its most recent report in November 2021, estimated that 37.7 million people globally are living with HIV. It was also reported that about 1.5 million new HIV infections and 680,000 AIDS-related deaths have occurred in the year 2020 [1,2,3,4]. In South Africa, approximately 8.2 million people were living with HIV in the year 2021 [4]. According to the South African mid-year population statistics 2021, there has been an unprecedented increase from 79,420 to 85,154 HIV-AIDS-related deaths in 2021 [4]. Recently, the easy availability of antiretrovirals (ARVs) has tremendously changed the pattern of death. ARVs have also helped prolong the lifespan of HIV-infected people in South Africa. Globally, about 27.5 million HIV-infected persons had access to ARVs in 2020, while approximately 5.6 million infected South Africans accessed ARVs in 2020 [1,4,5].

The highly active antiretroviral therapy (HAART) entails combining three or more antiretroviral drugs that are subject to the HIV-infected person’s viral load. HAART assists in regulating viral loads and preventing the progression to AIDS. Despite its success in prolonging HIV-infected patients’ lifespans, the use of HAART promotes metabolic syndrome (MetS) through an inflammatory pathway, excess production of reactive oxygen species (ROS), and mitochondrial dysfunction. Over three decades since its discovery, HAART has significantly improved the diagnosis and management of persons with HIV [6,7,8,9,10,11,12]. The persistence of MetS before and during HAART treatment further highlights the need for more studies on MetS inhibitory compounds, such as *Spirulina platensis* (SP) [13], *Moringa oleifera* [14,15], Curcumin [16], and Mangiferin [17]. However, there are other alternatives to combat MetS and these include exercise training [18], life style changes, and properly balanced healthy food choices [19,20].

SP is a blue-green microalgae commonly used as a traditional food by some Mexican and African people [21,22]. SP is mostly found in the alkaline water of volcanic lakes. In addition to its popular nutritional value, SP possesses various medicinal properties. It can induce both the humoral and cellular mechanisms of the immune system when consumed [22]. Interestingly, SP was linked with MetS-lowering properties, such as hypoglycemia [23], hypolipidemia [24], and hypotension [13]. Studies in rodents suggested that SP is particularly useful in preventing MetS [13]. SP contains oxidative stress inhibitors, phycocyanin and phycocyanobilin [25,26,27]. Previous studies have also demonstrated that SP inhibits oxidative stress [21,25,28,29] and promotes mitochondrial health [30,31,32,33], thereby inhibiting inflammation [25,34]. Furthermore, SP can prevent the development of atherosclerosis [28] and diabetes [25]. The Food and Agriculture Organization (FAO) of the United Nations recommends *Spirulina* as a daily dietary supplement [35]. Microalgaes including *Spirulina* are environmentally friendly and have a high rate of yield in large-scale production under controlled conditions [36]. *Spirulina*, in addition to its nutritional and medicinal security, has the potential to eliminate poverty. The considerable potential for sustainable financial development in a small-scale crop for nutritional enhancement was evident in China, where the production increase resulted in a dramatic increase in profit from USD 7.6 million to USD 16.6 million. *Spirulina* production is possible for small and marginal farmers as well as enthusiastic urban gardeners; this makes it easily accessible to the population [37]. Moreover, the health system can provide SP as medication to control adverse effects in people living with HIV on HAART with minimal costs.

Due to the increasing number of HIV-infected people and their high dependence on HAART, it is imperative to explore the anti-inflammatory and antioxidant mechanisms of SP against HIV and MetS. This review explores the anti-inflammatory and antioxidant mechanisms of SP in the inhibition of MetS and its potential as a supplement against HIV and ARV-induced MetS.

## 2. The Roles of HIV and HAART in MetS

HIV has often been associated with MetS [38], which results in cardiovascular diseases. Recent reports have linked some HIV-related features to MetS. These characteristic features include escalated cases of cardiovascular diseases, type 2 diabetes mellitus, dyslipidemia, immunodeficiency, high viral load, and atherosclerosis [7]. Recently, studies have suggested that HAART, in addition to the above-mentioned HIV-related features, actively induces MetS in persons with HIV [7,39,40,41]. Earlier studies by Palios et al. (2011) on arterial stiffness, displayed by pulse wave velocity (PWV), and markers of MetS, reported that persons with HIV exhibited an increased degree of PWV when compared with the healthy controls. Subsequently, persons on HAART have been shown to exhibit a similar PWV as persons with hypertension [39,40]. The prevalence of MetS in HIV-infected people receiving antiretroviral (ARV) treatment was higher when compared to the general population. This prevalence was attributed to age, physical inactivity, and a low cluster of differentiation 4 (CD4) count [42,43,44]. The patient response to HAART varies; some antiviral drugs can successfully suppress the plasma viral load without increasing the CD4 count; this allows the risks of opportunistic infections and abnormalities. Failure to increase the CD4 count during HAART may be due to several factors, including drug resistance, low CD4+ T-cell count at the initiation of HAART, the advanced stage of the disease, and a low adherence to HAART [45].

The nuclear factor-kappa-light chain-enhancer of the activated B cell (NF-κB) is a protein complex that is responsible for DNA and cytokine (IL-1β, IL-6, and TNF-α) transcription, chemokine activation, and the survival of cells [7]. NF-κB is also a well-known mediator of inflammation that promotes HIV transcription [7,46].

Inflammation linked to HAART is evidenced by the persistently high levels of interleukin 6 (IL-6), C-reactive proteins, and D-dimers [47]. HIV creates chronic pro-inflammatory conditions that promote MetS [7]. Studies have shown that HIV is associated with inflammation, apoptosis, and mitochondrial dysfunction [7,48]; however, the mechanism that links HIV with MetS remains unclear [7]. Herein, we highlight the significance of SP as an anti-inflammatory supplement for HIV-infected people on lifelong HAART and its mechanism of inhibition on MetS.

## 3. Spirulina Species

*Spirulina* has three commonly investigated species due to their potential therapeutic nature and high nutritional content. These *Spirulina* species include *Spirulina platensis* (SP) (otherwise known as *Arthrospira platensis*), *Spirulina maxima* (*Arthrospira maxima*), and *Spirulina fusiformis* (*Arthrospira fusiformis*). These *Spirulina* species are also classified as oxygenic photosynthetic bacteria under Cyanobacteria and Prochlorales [49,50,51,52,53,54]. SP is found in alkaline water with abundant bicarbonate and saline [22,55]. *Spirulina* species are generally three-dimensional helix microstructures [56] protected by a cell wall composed of complex sugars and proteins [22]; however, helical transformation results after mature trichomes divide into hormogonia, binary fission, and undergo length elongation [57]. SP is considered an antioxidant and anti-inflammatory agent [58]. It is also considered as a nutraceutical food supplement due to its high content of proteins, vitamins, and minerals. Moreover, its composition includes chlorophyll, phycocyanin, and carotenoid. Chlorophyll has antioxidant and antimutagenic properties [59,60], carotenoids are vitally important antioxidants with cancer-inhibiting abilities [53], and phycocyanin is a Bili protein with antioxidant and radical scavenging properties [61]. Moreso, SP has also been credited for its cancer- and viral infection-suppressing abilities [62]. 

### 3.1. Role of Spirulina in the Inhibition of Oxidative Stress

**Figure 1:**
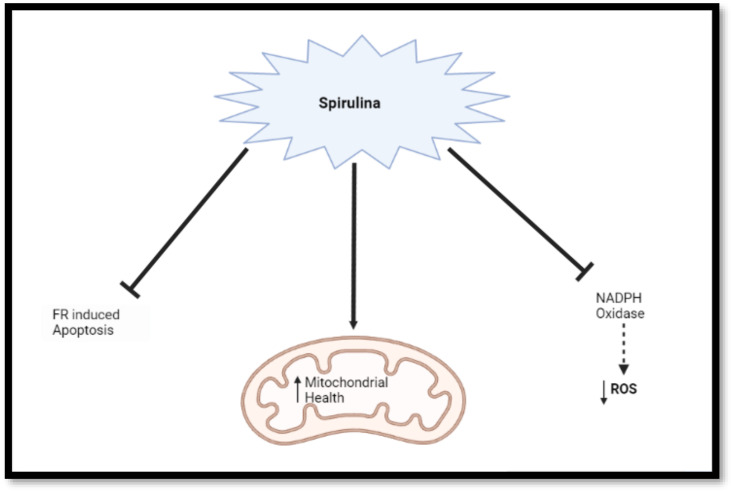
Diagrammatic representation of *Spirulina* reduction of oxidative stress via several pathways. *Spirulina* inhibits NADPH oxidase, reduces ROS, blocks FR-induced apoptosis, and promotes mitochondrial health (Created with BioRender.com, accessed on 17 November 2021).

Recently, research has unveiled the important roles of nicotinamide adenine dinucleotide phosphate (NADPH) oxidase in the production of reactive oxygen species (ROS). NADPH is contained in the nervous system and its assemblage and activation generate free radicals (FR) which subsequently destroy cells. *Spirulina* is a potent inhibitor of NADPH oxidase, which has been one of the proteins responsible for the production of ROS and subsequent oxidative stress [54,63,64]. SP has the potential to inhibit oxidative stress by blocking NADPH oxidase [21,25,28,29] and to enhance (Figure 1) mitochondrial health by promoting an antioxidant response [30,31,32,33]. Furthermore, SP prevents FR-induced apoptotic cell death through the inhibition of oxidative stress [65].

### 3.2. Multitargeted Therapeutic Roles of SP

**Figure 2:**
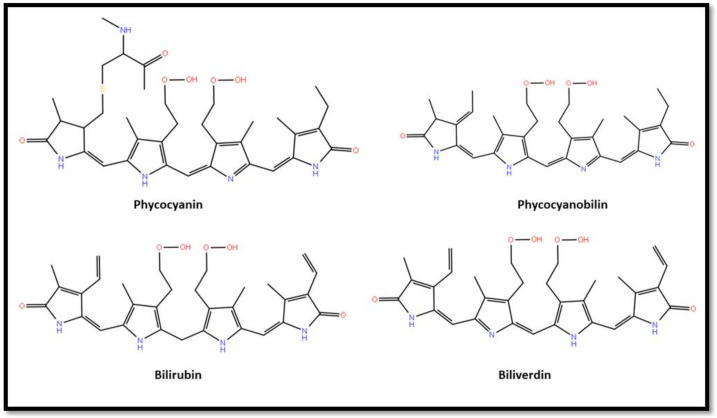
The 2D chemical structures of phycocyanin, phycocyanobilin, bilirubin, and biliverdin (prepared by author using maestro 11.2).

SP has a therapeutic effect against vascular diseases, cancer, diabetes, neurodegenerative diseases, and inflammatory disorders [66]. *Spirulina* treatment enhances the NF-kappa B-directed luciferase expression [67]. It has antiallergic effects [54], including against allergic rhinitis in humans [68]. *Spirulina* is an immune booster [22]. It prevents cellular aging, infectious diseases, and promotes a strong immune system [57]. *Spirulina* has central neuroprotective effects in rodents [69]. It is also associated with the inhibitory effects against numerous viruses, such as HIV-1, herpes simplex virus 1 and 2 (HSV-1 and HSV-2), human cytomegalovirus (HCMV), influenza type A, measles, and other enveloped viruses [53,70,71,72,73]. Moreover, it has antimutagenic and anticancer effects [57]. *Spirulina* is an effective treatment against chronic arsenic poisoning with melanosis and keratosis [74]. SP shares similar chemical structures and physiological activities with bilirubin [25,26,27,28,75]. SP antioxidant properties are due to its composition and the presence of phycobilins, phycocyanin, and phycocyanobilin [25,26,27] (Figure 2). Phycobilins are similar in structure to bile pigments such as bilirubin, a well-known ROS scavenger [22,76]. Phycocyanin has been proven to possess antioxidant and anti-inflammatory activities [34,54,63,64,77,78]. Phycocyanin is also structurally similar to biliverdin, a strong inhibitor of NADPH oxidase and inflammation-induced radicals [25,34]. A study conducted by Zheng et al. (2013) indicated that phycocyanin normalized urinary and renal oxidative stress markers and the expression of NADPH oxidase components. Furthermore, phycocyanobilin, bilirubin, and biliverdin inhibited NADPH-dependent superoxide production in renal mesangial cells [25]. The study also demonstrated that SP may be used in a therapeutic approach to prevent diabetic nephropathy through the inhibition of oxidative stress [25]. Thus far, phycocyanine, the most powerful natural antioxidant, is only present in cyanobacteria and thus in *spirulina*.

**Figure 3:**
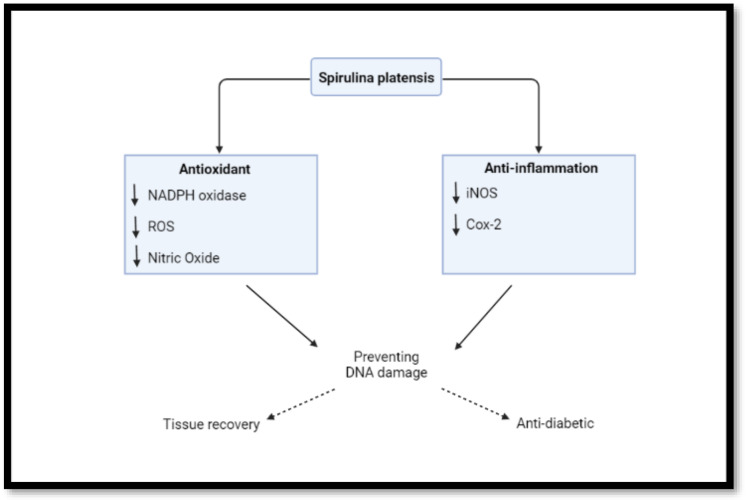
Antioxidant and anti-inflammatory effects of SP (created with BioRender.com, accessed on 17 November 2021).

SP exhibited neuroprotective activities through antioxidant and anti-inflammatory effects [79]. It has also shown significant antioxidant activity in vitro by scavenging nitric oxide and preventing DNA damage by scavenging hydroxyl radical (Figure 3) [80]. Antidiabetic and anti-inflammatory properties of SP [81] are based on its significant free-radical scavenging activities. SP contains compounds that fall under a broad spectrum of antioxidant agents, such as alkaloids, flavonoids [82], and phycocyanin [28,83,84,85,86]. Moreso, it provides trace minerals for the synthesis of antioxidant enzymes, demonstrated by the antidiabetic response in rats [87]. It has potential benefits in assisting with the reduction in chronic inflammatory conditions [88]. *Spirulina* incorporated into skin cream showed promising results as an anti-inflammatory and a wound-healing agent [89].

*Spirulina* against HIV-1 demonstrated its ability as an antiviral compound. Studies have demonstrated the ability of SP in the inhibition of HIV-1 replication in human T-cell lines, peripheral blood mononuclear cells (PBMC), and Langerhans cells (LC). The inhibition of the viral production by *spirulina* extract (between 0.3 and 1.2 μg/mL) was found to be approximately 50% in PBMCs [57]. More studies are needed to fully understand the mechanism behind the inhibition of HIV by SP.

### 3.3. Mechanism of Action

SP contains several vital antioxidant and anti-inflammatory compounds as mentioned above, such as chlorophyll, phycocyanin, and carotenoids (β-carotene). The antioxidant and anti-inflammatory properties of phycocyanin have been determined in numerous studies [28,83,84,85,86,90,91,92,93,94,95,96,97]. Phycocyanin is responsible for reducing oxidative stress and NADPH oxidase [28]. It scavenges free radicals, such as alkoxy, hydroxyl, and peroxyl radicals, and decreases nitrite production and inducible nitric oxide synthase (iNOS) expression. Phycocyanin also inhibits liver microsomal lipid peroxidation [28,83,84,85,86,90,91,92,93,94,95,96,97]. 

Phycocyanin has been proven to inhibit the formation of the pro-inflammatory cytokine TNF-α and cyclooxygenase-2 (COX-2) expression. Additionally, it decreases prostaglandin E(2) production [28,83,84,85,86]. Phycocyanin prevents the degradation of cytosolic IκB-α, which suppresses the activation of nuclear factor-κB (NF-κB) [28]. Furthermore, the inhibitory activity of phycocyanin is associated with the suppression of TNF-α formation in the macrophages [86]. In addition, phycocyanin exerts regulatory effects on mitogen-activated protein kinase (MAPK) activation pathways, such as the p38, c-Jun N-terminal kinase (JNK), and extracellular-signal-regulated kinase (ERK1/2) pathways [98,99,100]. The second compound of SP, carotenoids, β-carotene to be specific, is an antioxidant that has anti-carcinogenic, antioxidant, and anti-inflammatory activities [101,102,103]. As a membrane antioxidant, β-carotene protects against singlet oxygen-mediated lipid peroxidation [101]. Beta carotene inhibits the production of nitric oxide and prostaglandin E(2) and suppresses the expression of inducible nitric oxide synthase (iNOS), COX-2, TNF-α, and IL-1β. The suppression of inflammatory mediators by β-carotene results from its’ ability to inhibit NF-κB activation by preventing nuclear translocation of the NF-κB p65 subunit [102,104,105]. Studies have shown that β-carotene suppressed the transcription of inflammatory cytokines such as IL-1β, IL-6, and IL-12 in vitro [103]; this takes place in the macrophages. The third compound of *spirulina*, chlorophyll, can perform antioxidant and antimutagenic activities [59,60]. *Spirulina*’s mechanism of action is a concert of compounds, but it is not clear whether they all act simultaneously during demanding events. 

SP promotes the activation and expression of heme oxygenase 1 (HO-1) and endothelial nitric oxide synthase (eNOS) [104,105]. HO-1 is suggested to play an important part in the adaptive reprogramming which could result in Nrf-2 activation, but this pathway is unclear, and more studies are required. Moreso, SP causes the activation of the Nrf2/HO-1 pathway [106]. Nrf-2 activation by SP results in the production and increased expression of antioxidant enzymes, such as superoxide dismutase (SOD) and catalase (CAT) [100]. 

## 4. Common Highly Active Antiretroviral Therapy (HAART) Combinations

HAART has several classes: nucleoside/nucleotide reverse transcriptase inhibitors (NRTIs), non-nucleoside reverse transcriptase inhibitors (NNRTIs) [48,107,108,109,110,111,112,113,114], protease inhibitors (PIs) [114,115,116,117], integrase strand transfer inhibitors (INSTIs) [118,119,120,121,122,123], fusion inhibitors (FIs) [124], and chemokine receptor antagonists (CCR5 antagonists) [125,126,127]. HAART is a specifically selected combination of NRTI and NNRTI, PI, or INSTI drugs responsible for the inhibition of viral replication by multiple virus targets [113,128,129,130,131,132,133,134,135,136]. However, HAART can cause adverse drug reactions. Furthermore, 2′,3′-dideoxy-3′-thiacytidine (3TC), 2′,3′-dideoxy-5-fluoro-3′-thiacytidine (FTC), TDF (Tenofovir Disoproxil Fumarate), ZDV (Zidovudine), and d4T (Stavudine) are associated with mitochondrial toxicity and oxidative stress [137,138,139,140]. Additionally, NNRTIs are linked to toxic hepatitis, and PIs are implicated in insulin resistance and hyperlipidemia [114]. Chronic side effects linked to HAART include ROS-induced insulin resistance [141,142], lipodystrophy, gastrointestinal disorders [143], and cardiovascular disease [11,144]. Primarily, HAART is used for the treatment and prevention of HIV-1. These primary functions of HAART are achieved by attacking different components of the virus lifecycle which ensures inhibition regardless of the virus being resistant to one of the drugs [113,128,129,130,131,132,133,134,135,136]. A combination of two NRTIs (mostly FTC and TDF) and one NNRTI (e.g., EFV; Efavirenz) is a more favorable choice due to the convenience to dose, effectiveness, and less toxic effects compared to other drug combinations [114,145,146]. A combination of three NRTIs is less effective than two NRTIs with an NNRTI [147]. The d4T/ddI combination is associated with high toxicity and hence it is not often recommended [148]. The most popular NRTIs are cytidine analogs (XTC), FTC, 3TC, and TDF, which form part of the first-line therapy [149,150,151,152,153]. FTC and 3TC are similar in chemical structures with different pharmacokinetic and pharmacodynamic properties, and they have required deoxynucleosides for HIV DNA synthesis. They undergo phosphorylation through intracellular kinases to become FTC 50 -triphosphate (FTC-TP) and 3TC-TP; triphosphate metabolites with FTC-TP are more efficiently incorporated during HIV DNA synthesis than 3TC-TP [145,154,155,156,157]. Moreover, 3TC-TP has a shorter intracellular half-life compared to FTC-TP [155]. The TDF and FTC combination has a synergistic effect, increasing intracellular metabolites, and they are recommended for pre-exposure prophylaxis (PrEP) [155,158,159,160,161]. Contraindications (Table 1) on HAART treatment are antiretroviral medication-specific and can be overcome by a change in the HAART combination to suit the individuals own treatment [107,108,119,120,121,131,162,163,164,165] or the supplementation and treatment of the symptoms.

## 5. Mechanism of HAART-Induced Oxidative Stress, Inflammation, and Mitochondrial Dysfunction

The exact mechanism of HAART-induced oxidative stress has not been completely explored; however, studies have demonstrated the link between HAART use and oxidative stress. HAART is linked with lipid metabolism dysfunction through the induction of peripheral lipodystrophy. Lipodystrophy results from the impaired cytoplasmic retinoic-acid binding protein type 1 (CRABP1)-mediated cis-9-retinoic acid stimulation of peroxisome proliferator-activated receptor type gamma (PPAR-γ), leading to impaired differentiation and increased apoptosis of peripheral adipocytes. HIV-1 protease-inhibitors further inhibit the cytochrome P450 3A-mediated synthesis of cis-9-retinoic acid, one of the key activators of PPAR-γ [166]. Insulin resistance occurs following impaired fat storage and lipid release [141,142,166], which impacts the oxidant profile. The depletion of ATP production and mitochondrial dysfunction [8,9], and the depletion of mitochondrial DNA [167,168,169], are some of the ways HAART induces oxidative stress. HIV increases oxidative stress, and HAART increases lipid oxidation, which amplifies the ROS imbalance leading to increased oxidative stress complications [10,170]. 

Chronic exposure to HAART induces increased oxidative stress in endothelial cells and mononuclear cell recruitment, which leads to cardiovascular diseases in HIV patients on ARV therapy [12]. Inducing oxidative stress is common for protease and reverse transcriptase inhibitors [171]. HAART drugs induce oxidative stress in various ways, which include inhibiting DNA pol-γ activity and leading to mitochondrial dysfunction, and also through the depletion of mitochondrial DNA [167,168,169]. Studies have shown that patients on HAART have abnormally high levels of free oxygen radicals in sera compared to untreated HIV patients and HIV-uninfected participants [10,11,168,170,172,173]. 

Adverse drug reactions vary; TDF and lopinavir cause acute and chronic renal dysfunction [174,175,176,177,178]. TDF inhibits mitochondrial DNA-polymerase gamma, hence leading to the impaired function of energy-dependent transporters [179,180]. NRTIs are associated with the inhibition of mitochondrial DNA polymerase, lactic acidosis, subcutaneous lipoatrophy, peripheral neuropathy, and pancreatitis. The level of mitochondrial toxicity depends on the drugs; it is low with 3TC, FTC, and TDF, followed by ZDV, and higher with d4T. NNRTIs are associated with life-threatening skin reactions and toxic hepatitis. PIs are associated with insulin resistance and hyperlipidemia [7,114].

## 6. Combined and Synergistic Therapeutic Actions of HAART and SP

Studies have shown that the possible therapeutic effects of antioxidants may provide strategies in suppressing oxidative stress-induced comorbidities that emerge with the use of HAART therapy in HIV-infected individuals [12]. The combination of HIV and HAART has been associated with increased oxidative stress and lipid peroxidation. Furthermore, HIV or HAART induces ROS by inducing NADPH oxidase [181,182]. Interestingly, SP is a potent antioxidant [26,27] with anti-inflammatory activities [34], which makes it a potential supplement in the mitigation of oxidative stress induced by HAART adverse drug reactions. Moreover, SP can inhibit NADPH oxidase which is considered one of the main sources of ROS and free radicals in HIV-infected persons on HAART [34,181,182], resulting in reduced oxidative stress [28]. Moreover, β-carotene from SP protects against singlet oxygen-mediated lipid peroxidation [101]. Among HAART complications, TDF and lopinavir cause acute and chronic renal dysfunction [174,175,176,177,178]. Herein, phycocyanin from SP can normalize urinary and renal oxidative stress markers and inhibit NADPH-dependent superoxide production in renal mesangial cells [25], ameliorating renal dysfunction. Lately, SP has been an effective therapeutic approach to preventing diabetic nephropathy through the inhibition of oxidative stress [25]. These properties indicate SP as a potential agent to mitigate renal dysfunction caused by HAART. As mentioned above, NRTIs can inhibit mitochondrial DNA polymerase [179,180]. Studies in vitro showed that SP can enhance cell nucleus enzyme function, repair DNA synthesis [57], and enhance mitochondrial health [30,31,32,33,80]. Mitochondrial toxicity presented as peripheral neuropathy and lactic acidosis can be corrected by SP through providing trace minerals for the synthesis of antioxidant enzymes [87] and reducing chronic inflammatory conditions [88].

NNRTIs are associated with life-threatening skin reactions and toxic hepatitis [114], these conditions may be ameliorated by SP. Phycocyanin from SP can inhibit liver microsomal lipid peroxidation [28,83,84,85,86,90,91,92,93,94,95,96,97], and hence reducing toxic hepatitis. Moreso, SP incorporated into skin creams showed promising results as an anti-inflammatory and a wound-healing agent [89]; this can be beneficial in the mitigation of NNRTI-induced skin reactions. PI therapy induces insulin resistance and hyperlipidemia [7]. Additionally, HAART may be associated with a higher risk of myocardial infarction [114,183,184]. SP has a therapeutic effect against vascular diseases, cancer, diabetes, and neurodegenerative diseases [66]. In addition, the *Spirulina* family has also shown central neuroprotective effects in rodents [69] and may exert its neuroprotective activities through antioxidant and anti-inflammatory effects [79]. Therefore, SP is a recommended antioxidant to use as a supplement; the list of benefits is evident. It also has antiallergic effects [54], prevents cellular aging and infectious diseases, and promotes a strong immune system [57]. Herein, promotion of a strong immune system by SP can help increase CD4 cell counts, lower HIV viral loads, and slow down the progression to AIDS. Moreover, SP prevents FR-induced apoptotic cell death [65]; this may help decrease apoptosis of peripheral adipocytes induced by HAART. Chemically, SP is a recommended source of proteins, vitamins, and minerals [57], important nutrients for individuals on the HAART program. Finally, SP can assist HAART in the inhibition of HIV-1 replication because it has been shown to inhibit viral production in PBMCs.

There has been a number of clinical studies to investigate whether SP improves the quality of life in HIV-infected individuals. Marcel (2011) reported that insulin sensitivity in HIV patients improved more when a spirulina nutritional supplement was used instead of soybean [185]. Another study demonstrated for the first time that spirulina improves antioxidant capacity in people living with HIV [186]. *Spirulina* supplementation combined with a qualitative balanced diet showed potential to inhibit lipid abnormalities [187], significantly increase CD4 cells, and reduce the viral load in HIV-infected antiretroviral-naive patients [187,188,189]. However, there is limited information on the investigation of SP confirming the mechanism of antioxidant and anti-inflammatory effects and the impact on the quality of life in the HIV-positive population taking HAART. Thus far, it has been shown that supplementation with *Spirulina* platensis could improve the immune status of HIV patients on ART and decrease inflammatory and pro-oxidant levels [190]. The development of more clinical studies to confirm the SP protective effect in this population will answer many questions. The recommended concentrations of SP for daily supplementation varies, as studies have successfully used 19 g [185], 5 g [186], and 10 g [190,191].

## 7. Conclusions

HIV continues to be a major global cause of mortality. Besides the therapeutic benefits of HAART in HIV treatment, HAART has been linked to numerous adverse drug reactions which include oxidative stress, inflammation, and the disruption of mitochondrial function. SP as an antioxidant, anti-inflammatory, anticancer, and nutritional supplement possesses various corrective properties against attacks from viruses and bacteria. The corrective health properties of SP are largely attributed to antioxidant pigments found in SP. These pigments include chlorophyll, carotenoids, and phycocyanin which facilitate antioxidant, anti-inflammatory, and anticancer properties. The corrective properties of SP indicated in this review highlight its potential in the mitigation of HAART adverse drug reactions and MetS. The SP supplement potential is also supported by its ability to assist HAART in the inhibition of HIV-1. This review highlighted the corrective properties of potent antioxidant SP potential as a supplement for individuals on lifelong HAART experiencing MetS. Furthermore, this review highlights the need for more studies on SP and HAART synergy.

## Figures and Tables

**Table 1:** HAART drugs mechanism and their adverse effects.

HAART	Mechanism	Example	Adverse Effect
Nucleoside/Nucleotide reverse transcriptase inhibitors (NRTIs)	NRTIs require intracellular phosphorylation via host enzymes before they can inhibit viral replication. These agents are nucleoside or nucleotide analogs with an absent hydroxyl at the 3′ end that are incorporated into the growing viral DNA strand. They competitively bind to reverse transcriptase and cause premature DNA chain termination as they inhibit 3′ to 5′ phosphodiester bond formation.	abacavir, didanosine, lamivudine, stavudine, tenofovir, emtricitabine, atazanavir, and zidovudine	Mitochondrial toxicity, bone marrow suppression, anemia, and lipodystrophy. NRTIs inhibit mitochondrial DNA polymerase. Tenofovir may cause kidney injury or decreased bone mineral density or osteoporosis. Abacavir is associated with a CD8-mediated hypersensitivity reaction in patients with the HLA-B*5701 mutation. Didanosine is associated with a high risk of pancreatitis and hepatomegaly.
Non-nucleoside reverse transcriptase inhibitors (NNRTIs)	NNRTIs bind to HIV reverse transcriptase at an allosteric, hydrophobic site. These agents cause a stereochemical change within reverse transcriptase, thus inhibiting nucleoside binding and inhibition of DNA polymerase.	delavirdine, efavirenz, nevirapine, rilpivirine	Temporary rashes but may progress to Stevens–Johnson’s syndrome. Hepatitis may progress to liver failure. Efavirenz may cause teratogenicity Risk of neural tube defects. NNRTIs (mostly rilpivirine) may result in QT prolongation. Numerous interactions with hepatic cytochrome P450 enzymes. Efavirenz is linked to various psychiatric and CNS effects, including, but not limited to: vivid dreams, delusions, sleep disturbances, dizziness, headaches, increased suicidality, psychosis-like behavior, and mania.
Protease inhibitors (PIs)	PIs competitively inhibit the proteolytic cleavage of the gag/pol polyproteins in HIV-infected cells. These agents result in immature, noninfectious virions. PIs are administered with boosting agents such as ritonavir or cobicistat to patients that are failing their initial HAART combination.	atazanavir, darunavir, indinavir	Hepatotoxicity, insulin resistance, hyperglycemia, hyperlipidemia, lipodystrophy, and PR interval prolongation. Other PIs are inefficient and have a high resistance and increased risk of nephrolithiasis, hence indinavir and saquinavir are no longer used.
Integrase strand transfer inhibitors (INSTIs)	Integrase inhibitors bind viral integrase and prevent viral DNA from being incorporated into the host cell chromosome.	dolutegravir, elvitegravir, raltegravir	Some patients may experience dizziness, sleep disturbances, or depression. Raltegravir and dolutegravir can cause rhabdomyolysis and myopathy. Dolutegravir can block the secretion of creatinine and occasionally cause a decrease in the GFR. It can also have interactions with several medications, including those that inhibit/induce CYP3A4 enzymes, metformin, rifampin, and antiepileptics.
Fusion inhibitors (FIs)	Fusion inhibitors bind to the envelope glycoprotein gp41 and prevent viral fusion to the CD4 T-cells.	enfuvirtide	Enfuvirtide is generally well tolerated though some patients may experience injection site reactions.
Chemokine receptor antagonists (CCR5 antagonists)	CCR5 antagonists selectively and reversibly block entry into the CD4 T-cells by preventing interaction between CD4 cells and the gp120 subunit of the viral envelope glycoprotein.	maraviroc	Some patients may experience dizziness or skin rashes. There is a risk of hepatotoxicity with allergic features, high risk of hepatic dysfunction. Drug–drug interactions should be a consideration if patients are taking concurrent CYP3A4 inhibitors or inducers.

Abbreviations: HLA—human leucocyte antigen; CNS—central nervous system; GFR—glomerular filtration rate.

## Data Availability

Not applicable.

## References

[1] WHO HIV/AIDS. https://www.who.int/news-room/fact-sheets/detail/hiv-aids.

[2] UNAIDS (2021). Joint United Nations Programme on HIV/AIDS (UNAIDS). Global Report: UNAIDS Report on the Global AIDS Epidemic 2021.

[3] UNAIDS Global HIV & AIDS Statistics—2020 Fact. https://www.unaids.org/en/resources/fact-sheet.

[4] Release S. (2021). Mid-Year Population Estimates 2021. Statistics South Africa. http://www.statssa.gov.za/publications/P0302/P03022021.pdf.

[5] UNAIDS HIV and AIDS Estimates. https://www.unaids.org/en/regionscountries/countries/southafrica.

[6] National Institute on Drug Abuse (NIDA) What Is HAART?. https://www.drugabuse.gov/publications/research-reports/hivaids/what-haart.

[7] Mohan J., Ghazi T., Chuturgoon A.A. (2021). A Critical Review of the Biochemical Mechanisms and Epigenetic Modifications in HIV- and Antiretroviral-Induced Metabolic Syndrome. Int. J. Mol. Sci..

[8] Manda K.R., Banerjee A., Banks W.A., Ercal N. (2011). Highly active antiretroviral therapy drug combination induces oxidative stress and mitochondrial dysfunction in immortalized human blood–brain barrier endothelial cells. Free Radic. Biol. Med..

[9] Blas-Garcia A., Apostolova N., Esplugues J.V. (2011). Oxidative stress and mitochondrial impairment after treatment with anti-HIV drugs: Clinical implications. Curr. Pharm. Des..

[10] Ngondi J.L., Oben J., Forkah D.M., Etame L.H., Mbanya D. (2006). The effect of different combination therapies on oxidative stress markers in HIV infected patients in Cameroon. AIDS Res. Ther..

[11] Masiá M., Padilla S., Bernal E., Almenar M.V., Molina J., Hernández I., Graells M.L., Gutiérrez F. (2007). Influence of antiretroviral therapy on oxidative stress and cardiovascular risk: A prospective cross-sectional study in HIV-infected patients. Clin. Ther..

[12] Mondal D., Pradhan L., Ali M., Agrawal K.C. (2004). HAART drugs induce oxidative stress in human endothelial cells and increase endothelial recruitment of mononuclear cells. Cardiovasc. Toxicol..

[13] Finamore A., Palmery M., Bensehaila S., Peluso I. (2017). Antioxidant, immunomodulating, and microbial-modulating activities of the sustainable and ecofriendly spirulina. Oxidative Med. Cell. Longev..

[14] Nyakudya T.T., Tshabalala T., Dangarembizi R., Erlwanger K.H., Ndhlala A.R. (2020). The potential therapeutic value of medicinal plants in the management of metabolic disorders. Molecules.

[15] Kim D.S., Choi M.H., Shin H.J. (2020). Extracts of Moringa oleifera leaves from different cultivation regions show both antioxidant and antiobesity activities. J. Food Biochem..

[16] Pérez-Torres I., Ruiz-Ramírez A., Baños G., El-Hafidi M. (2013). Hibiscus sabdariffa Linnaeus (Malvaceae), curcumin and resveratrol as alternative medicinal agents against metabolic syndrome. Cardiovasc. Hematol. Agents Med. Chem..

[17] Mujawdiya P.K., Kapur S. (2015). Mangiferin: A potential natural molecule for management of metabolic syndrome. Int. J. Pharm. Pharm. Sci..

[18] Kränkel N., Bahls M., van Craenenbroeck E.M., Adams V., Serratosa L., Solberg E.E., Hansen D., Dörr M., Kemps H. (2019). Exercise training to reduce cardiovascular risk in patients with metabolic syndrome and type 2 diabetes mellitus: How does it work?. Eur. J. Prev. Cardiol..

[19] Opie L.H. (2007). Metabolic syndrome. Circulation.

[20] Marrone G., Guerriero C., Palazzetti D., Lido P., Marolla A., Di Daniele F., Noce A. (2021). Vegan diet health benefits in metabolic syndrome. Nutrients.

[21] Bashandy S.A., El Awdan S.A., Ebaid H., Alhazza I.M. (2016). Antioxidant potential of Spirulina platensis mitigates oxidative stress and reprotoxicity induced by sodium arsenite in male rats. Oxidative Med. Cell. Longev..

[22] Estrada J.P., Bescós P.B., del Fresno A.V. (2001). Antioxidant activity of different fractions of Spirulina platensis protean extract. Il Farm..

[23] Iyer Uma M., Sophia A., Uliyar V.M. (1999). Glycemic and lipemic responses of selected spirulina-supplemented rice-based recipes in normal subjects. Age Years.

[24] Serban M.C., Sahebkar A., Dragan S., Stoichescu-Hogea G., Ursoniu S., Andrica F., Banach M. (2016). A systematic review and meta-analysis of the impact of Spirulina supplementation on plasma lipid concentrations. Clin. Nutr..

[25] Zheng J., Inoguchi T., Sasaki S., Maeda Y., McCarty M.F., Fujii M., Ikeda N., Kobayashi K., Sonoda N., Takayanagi R. (2013). Phycocyanin and phycocyanobilin from Spirulina platensis protect against diabetic nephropathy by inhibiting oxidative stress. Am. J. Physiol. -Regul. Integr. Comp. Physiol..

[26] Hu Z., Liu Z. (2001). Determination and purification of beta-carotene in Spirulina maximum. Chin. J. Chromatogr..

[27] Miranda M.S., Cintra R.G., Barros S., Mancini-Filho J. (1998). Antioxidant activity of the microalga Spirulina maxima. Braz. J. Med. Biol. Res..

[28] Riss J., Décordé K., Sutra T., Delage M., Baccou J.C., Jouy N., Brune J.P., Oréal H., Cristol J.P., Rouanet J.M. (2007). Phycobiliprotein C-phycocyanin from Spirulina platensis is powerfully responsible for reducing oxidative stress and NADPH oxidase expression induced by an atherogenic diet in hamsters. J. Agric. Food Chem..

[29] Abdelkhalek N.K., Ghazy E.W., Abdel-Daim M.M. (2015). Pharmacodynamic interaction of Spirulina platensis and deltamethrin in freshwater fish Nile tilapia, Oreochromis niloticus: Impact on lipid peroxidation and oxidative stress. Environ. Sci. Pollut. Res..

[30] Nawrocka D., Kornicka K., Śmieszek A., Marycz K. (2017). Spirulina platensis improves mitochondrial function impaired by elevated oxidative stress in adipose-derived mesenchymal stromal cells (ASCs) and intestinal epithelial cells (IECs), and enhances insulin sensitivity in equine metabolic syndrome (EMS) horses. Mar. Drugs.

[31] Jadaun P., Yadav D., Bisen P.S. (2018). Spirulina platensis prevents high glucose-induced oxidative stress mitochondrial damage mediated apoptosis in cardiomyoblasts. Cytotechnology.

[32] Sun J.Y., Hou Y.J., Fu X.Y., Fu X.T., Ma J.K., Yang M.F., Sun B.L., Fan C.D., Oh J. (2019). Selenium-containing protein from selenium-enriched spirulina platensis attenuates cisplatin-induced apoptosis in MC3T3-E1 Mouse preosteoblast by inhibiting mitochondrial dysfunction and ROS-Mediated oxidative damage. Front. Physiol..

[33] Oriquat G.A., Ali M.A., Mahmoud S.A., Eid R.M., Hassan R., Kamel M.A. (2019). Improving hepatic mitochondrial biogenesis as a postulated mechanism for the antidiabetic effect of Spirulina platensis in comparison with metformin. Appl. Physiol. Nutr. Metab..

[34] Izadi M., Fazilati M. (2018). Extraction and purification of phycocyanin from spirulina platensis and evaluating its antioxidant and anti-inflammatory activity. Asian J. Green Chem..

[35] Pelizer L.H., Danesi E.D.G., de O Rangel C., Sassano C.E., Carvalho J.C.M., Sato S., Moraes I.O. (2003). Influence of inoculum age and concentration in Spirulina platensis cultivation. J. Food Eng..

[36] Pina-Pérez M.C., Brück W.M., Brück T., Beyrer M. (2019). Microalgae as healthy ingredients for functional foods. The Role of Alternative and Innovative Food Ingredients and Products in Consumer Wellness.

[37] Uddin A.J., Mahbuba S., Rahul S., Ifaz M., Ahmad H. (2018). Super food Spirulina (Spirulina platensis): Prospect and scopes in Bangladesh. Int. J. Bus. Soc. Sci. Res..

[38] Li Vecchi V., Maggi P., Rizzo M., Montalto G. (2014). The metabolic syndrome and HIV infection. Curr. Pharm. Des..

[39] Aounallah M., Dagenais-Lussier X., El-Far M., Mehraj V., Jenabian M.A., Routy J.P., van Grevenynghe J. (2016). Current topics in HIV pathogenesis, part 2: Inflammation drives a Warburg-like effect on the metabolism of HIV-infected subjects. Cytokine Growth Factor Rev..

[40] Palios J., Kadoglou N.P., Lampropoulos S. (2011). The pathophysiology of HIV-/HAART-related metabolic syndrome leading to cardiovascular disorders: The emerging role of adipokines. Exp. Diabetes Res..

[41] Barbaro G., Iacobellis G. (2009). Metabolic syndrome associated with HIV and highly active antiretroviral therapy. Curr. Diabetes Rep..

[42] Rogalska-Płońska M., Grzeszczuk A., Rogalski P., Łucejko M., Flisiak R. (2018). Metabolic syndrome in HIV infected adults in Poland. Kardiol. Pol. Pol. Heart J..

[43] Nguyen K.A., Peer N., Mills E.J., Kengne A.P. (2016). A meta-analysis of the metabolic syndrome prevalence in the global HIV-infected population. PLoS ONE.

[44] Ataro Z., Ashenafi W. (2020). Metabolic syndrome and associated factors among adult HIV positive people on antiretroviral therapy in Jugal hospital, Harar, Eastern Ethiopia. East Afr. J. Health Biomed. Sci..

[45] Aiuti F., Mezzaroma I. (2006). Failure to reconstitute CD4+ T-cells despite suppression of HIV replication under HAART. AIDS Rev..

[46] Baker R.G., Hayden M.S., Ghosh S. (2011). NF-κB, inflammation, and metabolic disease. Cell Metab..

[47] Nasi M., De Biasi S., Gibellini L., Bianchini E., Pecorini S., Bacca V., Guaraldi G., Mussini C., Pinti M., Cossarizza A. (2017). Ageing and inflammation in patients with HIV infection. Clin. Exp. Immunol..

[48] Lucas G.M., Ross M.J., Stock P.G., Shlipak M.G., Wyatt C.M., Gupta S.K., Atta M.G., Wools-Kaloustian K.K., Pham P.A., Bruggeman L.A. (2014). Clinical practice guideline for the management of chronic kidney disease in patients infected with HIV: 2014 update by the HIV Medicine Association of the Infectious Diseases Society of America. Clin. Infect. Dis..

[49] Turner S. (1997). Molecular systematics of oxygenic photosynthetic bacteria. Orig. Algae Plast..

[50] Whitton B.A. (1992). Diversity, ecology, and taxonomy of the cyanobacteria. Photosynthetic Prokaryotes.

[51] Vonshak A. (1997). Spirulina Platensis Arthrospira: Physiology, Cell-Biology and Biotechnology.

[52] Gershwin M.E., Belay A. (2007). Spirulina in Human Nutrition and Health.

[53] Khan Z., Bhadouria P., Bisen P. (2005). Nutritional and therapeutic potential of Spirulina. Curr. Pharm. Biotechnol..

[54] Karkos P.D., Leong S.C., Karkos C.D., Sivaji N., Assimakopoulos D.A. (2011). Spirulina in clinical practice: Evidence-based human applications. Evid.-Based Complementary Altern. Med..

[55] Ciferri O. (1983). Spirulina, the edible microorganism. Microbiol. Rev..

[56] Kamata K., Piao Z., Suzuki S., Fujimori T., Tajiri W., Nagai K., Iyoda T., Yamada A., Hayakawa T., Ishiwara M. (2014). Spirulina-templated metal microcoils with controlled helical structures for THz electromagnetic responses. Sci. Rep..

[57] Ali S.K., Saleh A.M. (2012). Spirulina-an overview. Int. J. Pharm. Pharm. Sci..

[58] Henrikson R. (1989). Earth Food Spirulina.

[59] Ferruzzi M.G., Blakeslee J. (2007). Digestion, absorption, and cancer preventative activity of dietary chlorophyll derivatives. Nutr. Res..

[60] Hosikian A., Lim S., Halim R., Danquah M.K. (2010). Chlorophyll extraction from microalgae: A review on the process engineering aspects. Int. J. Chem. Eng..

[61] Hoseini S.M., Khosravi-Darani K., Mozafari M.R. (2013). Nutritional and medical applications of spirulina microalgae. Mini Rev. Med. Chem..

[62] Asghari A., Fazilati M., Latifi A.M., Salavati H., Choopani A. (2016). A review on antioxidant properties of Spirulina. J. Appl. Biotechnol. Rep..

[63] Wu Q., Liu L., Miron A., Klímová B., Wan D., Kuča K. (2016). The antioxidant, immunomodulatory, and anti-inflammatory activities of Spirulina: An overview. Arch. Toxicol..

[64] Deng R., Chow T.J. (2010). Hypolipidemic, antioxidant, and antiinflammatory activities of microalgae Spirulina. Cardiovasc. Ther..

[65] Chu W.L., Lim Y.W., Radhakrishnan A.K., Lim P.E. (2010). Protective effect of aqueous extract from Spirulina platensis against cell death induced by free radicals. BMC Complementary Altern. Med..

[66] McCarty M.F. (2007). ‘‘Iatrogenic Gilbert syndrome’’–A strategy for reducing vascular and cancer risk by increasing plasma unconjugated bilirubin. Med. Hypotheses.

[67] Balachandran P., Pugh N.D., Ma G., Pasco D.S. (2006). Toll-like receptor 2-dependent activation of monocytes by Spirulina polysaccharide and its immune enhancing action in mice. Int. Immunopharmacol..

[68] Mao T., Water J.V.d., Gershwin M.E. (2005). Effects of a Spirulina-based dietary supplement on cytokine production from allergic rhinitis patients. J. Med. Food.

[69] Chamorro G., Pérez-Albiter M., Serrano-García N., Mares-Sámano J.J., Rojas P. (2006). Spirulina maxima pretreatment partially protects against 1-methyl-4-phenyl-1, 2, 3, 6-tetrahydropyridine neurotoxicity. Nutr. Neurosci..

[70] Hernández-Corona A., Nieves I., Meckes M., Chamorro G., Barron B.L. (2002). Antiviral activity of Spirulina maxima against herpes simplex virus type 2. Antivir. Res..

[71] Luescher-Mattli M. (2003). Algae, a possible source for new drugs in the treatment of HIV and other viral diseases. Curr. Med. Chem. -Anti-Infect. Agents.

[72] Hayashi T., Hayashi K., Maeda M., Kojima I. (1996). Calcium spirulan, an inhibitor of enveloped virus replication, from a blue-green alga Spirulina platensis. J. Nat. Prod..

[73] Ayehunie S., Belay A., Baba T.W., Ruprecht R.M. (1998). Inhibition of HIV-1 replication by an aqueous extract of Spirulina platensis (Arthrospira platensis). J. Acquir. Immune Defic. Syndr. Hum. Retrovirol..

[74] Choudhary M., Jetley U.K., Khan M.A., Zutshi S., Fatma T. (2007). Effect of heavy metal stress on proline, malondialdehyde, and superoxide dismutase activity in the cyanobacterium Spirulina platensis-S5. Ecotoxicol. Environ. Saf..

[75] Jiang F., Roberts S.J., Datla S.R., Dusting G.J. (2006). NO modulates NADPH oxidase function via heme oxygenase-1 in human endothelial cells. Hypertension.

[76] Bhat V.B., Madyastha K. (2000). C-phycocyanin: A potent peroxyl radical scavenger in vivo and in vitro. Biochem. Biophys. Res. Commun..

[77] Ashaolu T.J., Samborska K., Lee C.C., Tomas M., Capanoglu E., Tarhan Ö., Taze B., Jafari S.M. (2021). Phycocyanin, a super functional ingredient from algae; properties, purification characterization, and applications. Int. J. Biol. Macromol..

[78] Grover P., Bhatnagar A., Kumari N., Bhatt A.N., Nishad D.K., Purkayastha J. (2021). C-Phycocyanin-a novel protein from Spirulina platensis-In vivo toxicity, antioxidant and immunomodulatory studies. Saudi J. Biol. Sci..

[79] Lima F.A.V., Joventino I.P., Joventino F.P., de Almeida A.C., Neves K.R.T., do Carmo M.R., Leal L.K.A.M., de Andrade G.M., de Barros Viana G.S. (2017). Neuroprotective activities of Spirulina platensis in the 6-OHDA model of Parkinson’s disease are related to its anti-inflammatory effects. Neurochem. Res..

[80] Kamble S.P., Gaikar R.B., Padalia R.B., Shinde K.D. (2013). Extraction and purification of C-phycocyanin from dry Spirulina powder and evaluating its antioxidant, anticoagulation and prevention of DNA damage activity. J. Appl. Pharm. Sci..

[81] Joventino I.P., Alves H.G., Neves L.C., Pinheiro-Joventino F., Leal L.K.A., Neves S.A., Ferreira F.V., Brito G.A.C., Viana G.B. (2012). The microalga Spirulina platensis presents anti-inflammatory action as well as hypoglycemic and hypolipidemic properties in diabetic rats. J. Complementary Integr. Med..

[82] Anbarasan V., Kumar V.K., Kumar P.S., Venkatachalam T. (2011). In vitro evaluation of antioxidant activity of blue green algae Spirulina platensis. Int. J. Pharm. Sci. Res..

[83] Romay C., Delgado R., Remirez D., Gonzalez R., Rojas A. (2001). Effects of phycocyanin extract on tumor necrosis factor-α and nitrite levels in serum of mice treated with endotoxin. Arzneimittelforschung.

[84] Remirez D., Fernández V., Tapia G., González R., Videla L.A. (2002). Influence of C-phycocyanin on hepatocellular parameters related to liver oxidative stress and Kupffer cell functioning. Inflamm. Res..

[85] Patel A., Mishra S., Ghosh P. (2006). Antioxidant potential of C-phycocyanin isolated from cyanobacterial species *Lyngbya*, *Phormidium* and *Spirulina* spp.. Indian J. Biochem. Biophys..

[86] Cherng S.C., Cheng S.N., Tarn A., Chou T.C. (2007). Anti-inflammatory activity of c-phycocyanin in lipopolysaccharide-stimulated RAW 264.7 macrophages. Life Sci..

[87] Nasirian F., Dadkhah M., Moradi-Kor N., Obeidavi Z. (2018). Effects of Spirulina platensis microalgae on antioxidant and anti-inflammatory factors in diabetic rats. Diabetes Metab. Syndr. Obes. Targets Ther..

[88] Jensen G.S., Attridge V.L., Beaman J.L., Guthrie J., Ehmann A., Benson K.F. (2015). Antioxidant and anti-inflammatory properties of an aqueous cyanophyta extract derived from arthrospira platensis: Contribution to bioactivities by the non-phycocyanin aqueous fraction. J. Med. Food.

[89] Gunes S., Tamburaci S., Dalay M.C., Deliloglu Gurhan I. (2017). In vitro evaluation of Spirulina platensis extract incorporated skin cream with its wound healing and antioxidant activities. Pharm. Biol..

[90] Romay C.H., Armesto J., Remirez D., González R., Ledon N., Garcia I. (1998). Antioxidant and anti-inflammatory properties of C-phycocyanin from blue-green algae. Inflamm. Res..

[91] Romay C., Ledon N., Gonzalez R. (1998). Further studies on anti-inflammatory activity of phycocyanin in some animal models of inflammation. Inflamm. Res..

[92] Gonzalez R., Rodriguez S., Romay C., González A., Armesto J., Remirez D., Merino N. (1999). Anti-inflammatory activity of phycocyanin extract in acetic acid-induced colitis in rats. Pharmacol. Res..

[93] Remirez D., Ledon N., González R. (2002). Role of histamine in the inhibitory effects of phycocyanin in experimental models of allergic inflammatory response. Mediat. Inflamm..

[94] Romay C.H., Gonzalez R., Ledon N., Remirez D., Rimbau V. (2003). C-phycocyanin: A biliprotein with antioxidant, anti-inflammatory and neuroprotective effects. Curr. Protein Pept. Sci..

[95] Khan M., Varadharaj S., Shobha J.C., Naidu M.U., Parinandi N.L., Kutala V.K., Kuppusamy P. (2006). C-phycocyanin ameliorates doxorubicin-induced oxidative stress and apoptosis in adult rat cardiomyocytes. J. Cardiovasc. Pharmacol..

[96] Shih C.M., Cheng S.N., Wong C.S., Kuo Y.L., Chou T.C. (2009). Antiinflammatory and antihyperalgesic activity of C-phycocyanin. Anesth. Analg..

[97] Manconia M., Pendás J., Ledón N., Moreira T., Sinico C., Saso L., Fadda A.M. (2009). Phycocyanin liposomes for topical anti-inflammatory activity: In-vitro in-vivo studies. J. Pharm. Pharmacol..

[98] Khan M., Varadharaj S., Ganesan L.P., Shobha J.C., Naidu M.U., Parinandi N.L., Tridandapani S., Kutala V.K., Kuppusamy P. (2006). C-phycocyanin protects against ischemia-reperfusion injury of heart through involvement of p38 MAPK and ERK signaling. Am. J. Physiol. -Heart Circ. Physiol..

[99] Li X.L., Xu G., Chen T., Wong Y.S., Zhao H.L., Fan R.R., Gu X.M., Tong P.C., Chan J.C. (2009). Phycocyanin protects INS-1E pancreatic beta cells against human islet amyloid polypeptide-induced apoptosis through attenuating oxidative stress and modulating JNK and p38 mitogen-activated protein kinase pathways. Int. J. Biochem. Cell Biol..

[100] Sadek K.M., Lebda M.A., Nasr S.M., Shoukry M. (2017). Spirulina platensis prevents hyperglycemia in rats by modulating gluconeogenesis and apoptosis via modification of oxidative stress and MAPK-pathways. Biomed. Pharmacother..

[101] Schafer F.Q., Wang H.P., Kelley E.E., Cueno K.L., Buettner S.M.A.G. (2002). Comparing β-carotene, vitamin E and nitric oxide as membrane antioxidants. Biol. Chem..

[102] Bai S.K., Lee S.J., Na H.J., Ha K.S., Han J.A., Lee H., Kwon Y.G., Chung C.K., Kim Y.M. (2005). β-Carotene inhibits inflammatory gene expression in lipopolysaccharide-stimulated macrophages by suppressing redox-based NF-κB activation. Exp. Mol. Med..

[103] Katsuura S., Imamura T., Bando N., Yamanishi R. (2009). β-Carotene and β-cryptoxanthin but not lutein evoke redox and immune changes in RAW264 murine macrophages. Mol. Nutr. Food Res..

[104] Strasky Z., Zemankova L., Nemeckova I., Rathouska J., Wong R.J., Muchova L., Subhanova I., Vanikova J., Vanova K., Vitek L. (2013). Spirulina platensis and phycocyanobilin activate atheroprotective heme oxygenase-1: A possible implication for atherogenesis. Food Funct..

[105] Jensen G.S., Drapeau C., Lenninger M., Benson K.F. (2016). Clinical safety of a high dose of Phycocyanin-enriched aqueous extract from Arthrospira (Spirulina) platensis: Results from a randomized, double-blind, placebo-controlled study with a focus on anticoagulant activity and platelet activation. J. Med. Food.

[106] Piovan A., Battaglia J., Filippini R., Dalla Costa V., Facci L., Argentini C., Pagetta A., Giusti P., Zusso M. (2021). Pre-and Early Post-treatment With Arthrospira platensis (Spirulina) Extract Impedes Lipopolysaccharide-triggered Neuroinflammation in Microglia. Front. Pharmacol..

[107] Eggleton J.S., Nagalli S. (2020). Highly Active Antiretroviral Therapy (HAART).

[108] Chang J.L., Tsai A.C., Musinguzi N., Haberer J.E., Boum Y., Muzoora C., Bwana M., Martin J.N., Hunt P.W., Bangsberg D.R. (2018). Depression and suicidal ideation among HIV-infected adults receiving efavirenz versus nevirapine in Uganda: A prospective cohort study. Ann. Intern. Med..

[109] Lyseng-Williamson K.A., Reynolds N.A., Plosker G.L. (2005). Tenofovir disoproxil fumarate. Drugs.

[110] Chen Y.F., Stampley J.E., Irving B.A., Dugas T.R. (2019). Chronic nucleoside reverse transcriptase inhibitors disrupt mitochondrial homeostasis and promote premature endothelial senescence. Toxicol. Sci..

[111] Martin A.M., Nolan D., Gaudieri S., Almeida C.A., Nolan R., James I., Carvalho F., Phillips E., Christiansen F.T., Purcell A.W. (2004). Predisposition to abacavir hypersensitivity conferred by HLA-B * 5701 and a haplotypic Hsp70-Hom variant. Proc. Natl. Acad. Sci. USA.

[112] Kakuda T. (2000). Pharmacology of nucleoside and nucleotide reverse transcriptase inhibitor-induced mitochondrial toxicity. Clin. Ther..

[113] Günthard H.F., Saag M.S., Benson C.A., Del Rio C., Eron J.J., Gallant J.E., Hoy J.F., Mugavero M.J., Sax P.E., Thompson M.A. (2018). Antiretroviral drugs for treatment and prevention of HIV infection in adults: 2018 recommendations of the International Antiviral Society–USA Panel. JAMA.

[114] Yeni P. (2006). Update on HAART in HIV. J. Hepatol..

[115] Eastone J.A., Decker C.F. (1997). New-onset diabetes mellitus associated with use of protease inhibitor. Ann. Intern. Med..

[116] Soliman E.Z., Lundgren J., Roediger M.P., Duprez D.A., Temesgen Z., Bickel M., Shlay J.C., Somboonwit C., Reiss P., Stein J. (2011). Boosted protease inhibitors and the electrocardiographic measures of QT and PR durations. AIDS.

[117] Landovitz R.J., Ribaudo H.J., Ofotokun I., Na L.H., Godfrey C., Kuritzkes D.R., Sagar M., Brown T.T., Cohn S.E., McComsey G.A. (2014). Efficacy and tolerability of 3 nonnucleoside reverse transcriptase inhibitor–sparing antiretroviral regimens for treatment-naive volunteers infected with HIV-1: A randomized, controlled equivalence trial. Ann. Intern. Med..

[118] Gutiérrez F., Fulladosa X., Barril G., Domingo P. (2014). Renal tubular transporter-mediated interactions of HIV drugs: Implications for patient management. AIDS Rev..

[119] Hoffmann C., Welz T., Sabranski M., Kolb M., Wolf E., Stellbrink H.J., Wyen C. (2017). Higher rates of neuropsychiatric adverse events leading to dolutegravir discontinuation in women and older patients. HIV Med..

[120] Fettiplace A., Stainsby C., Winston A., Givens N., Puccini S., Vannappagari V., Hsu R., Fusco J., Quercia R., Aboud M. (2017). Psychiatric Symptoms in Patients Receiving Dolutegravir. JAIDS J. Acquir. Immune Defic. Syndr..

[121] Zash R., Makhema J., Shapiro R.L. (2018). Neural-Tube Defects with Dolutegravir Treatment from the Time of Conception. N. Engl. J. Med..

[122] Schafer J.J., E Squires K. (2010). Integrase Inhibitors: A Novel Class of Antiretroviral Agents. Ann. Pharmacother..

[123] Hazuda D.J., Felock P., Witmer M., Wolfe A., Stillmock K., Grobler J.A., Espeseth A., Gabryelski L., Schleif W., Blau C. (2000). Inhibitors of Strand Transfer That Prevent Integration and Inhibit HIV-1 Replication in Cells. Science.

[124] Hardy H., Skolnik P.R. (2004). Enfuvirtide, a New Fusion Inhibitor for Therapy of Human Immunodeficiency Virus Infection. Pharmacother. J. Hum. Pharmacol. Drug Ther..

[125] Dorr P., Westby M., Dobbs S., Griffin P., Irvine B., Macartney M., Mori J., Rickett G., Smith-Burchnell C., Napier C. (2005). Maraviroc (UK-427,857), a Potent, Orally Bioavailable, and Selective Small-Molecule Inhibitor of Chemokine Receptor CCR5 with Broad-Spectrum Anti-Human Immunodeficiency Virus Type 1 Activity. Antimicrob. Agents Chemother..

[126] Miao M., De Clercq E., Li G. (2020). Clinical significance of chemokine receptor antagonists. Expert Opin. Drug Metab. Toxicol..

[127] De Luca A., Pezzotti P., Boucher C., Döring M., Incardona F., Kaiser R., Lengauer T., Pfeifer N., Schülter E., Vandamme A.-M. (2019). Clinical use, efficacy, and durability of maraviroc for antiretroviral therapy in routine care: A European survey. PLoS ONE.

[128] Shafer R., Vuitton D. (1999). Highly active antiretroviral therapy (HAART) for the treatment of infection with human immunodeficiency virus type 1. Biomed. Pharmacother..

[129] Kitahata M.M., Koepsell T.D., Deyo R.A., Maxwell C.L., Dodge W.T., Wagner E.H. (1996). Physicians’ experience with the acquired immunodeficiency syndrome as a factor in patients’ survival. N. Engl. J. Med..

[130] Cunningham W.E., Tisnado D.M., Lui H.H., Nakazono T.T., Carlisle D.M. (1999). The effect of hospital experience on mortality among patients hospitalized with acquired immunodeficiency syndrome in California. Am. J. Med..

[131] Rackal J.M., Tynan A.-M., Handford C.D., Rzeznikiewiz D., Agha A., Glazier R. (2011). Provider training and experience for people living with HIV/AIDS. Cochrane Database Syst. Rev..

[132] Ford N., Migone C., Calmy A., Kerschberger B., Kanters S., Nsanzimana S., Mills E.J., Meintjes G., Vitoria M., Doherty M. (2018). Benefits and risks of rapid initiation of antiretroviral therapy. AIDS.

[133] Group I.S.S. (2015). Initiation of antiretroviral therapy in early asymptomatic HIV infection. N. Engl. J. Med..

[134] Thompson M.A., Aberg J.A., Hoy J.F., Telenti A., Benson C., Cahn P., Eron J.J., Günthard H.F., Hammer S.M., Reiss P. (2012). Antiretroviral treatment of adult HIV infection: 2012 recommendations of the International Antiviral Society–USA panel. Jama.

[135] Sax P.E., Tierney C., Collier A.C., Fischl M.A., Mollan K., Peeples L., Godfrey C., Jahed N.C., Myers L., Katzenstein D. (2009). Abacavir–lamivudine versus tenofovir–emtricitabine for initial HIV-1 therapy. N. Engl. J. Med..

[136] Post F.A., Moyle G.J., Stellbrink H.J., Domingo P., Podzamczer D., Fisher M., Norden A.G., Cavassini M., Rieger A., Khuong-Josses M.A. (2010). Randomized comparison of renal effects, efficacy, and safety with once-daily abacavir/lamivudine versus tenofovir/emtricitabine, administered with efavirenz, in antiretroviral-naive, HIV-1–infected adults: 48-week results from the ASSERT study. JAIDS J. Acquir. Immune Defic. Syndr..

[137] Glover M., Hebert V.Y., Nichols K., Xue S.Y., Thibeaux T.M., Zavecz J.A., Dugas T.R. (2014). Overexpression of mitochondrial antioxidant manganese superoxide dismutase (MnSOD) provides protection against AZT- or 3TC-induced endothelial dysfunction. Antivir. Res..

[138] Brown L., Jin J., Ferrell D., Sadic E., Obregon D., Smith A.J., Tan J., Giunta B. (2014). Efavirenz Promotes β-Secretase Expression and Increased Aβ1-40,42 via Oxidative Stress and Reduced Microglial Phagocytosis: Implications for HIV Associated Neurocognitive Disorders (HAND). PLoS ONE.

[139] Canale D., De Bragança A.C., Gonçalves J.G., Shimizu M.H.M., Sanches T.R., Andrade L., Volpini R.A., Seguro A.C. (2014). Vitamin D Deficiency Aggravates Nephrotoxicity, Hypertension and Dyslipidemia Caused by Tenofovir: Role of Oxidative Stress and Renin-Angiotensin System. PLoS ONE.

[140] Venhoff N., Setzer B., Melkaoui K., A Walker U. (2007). Mitochondrial Toxicity of Tenofovir, Emtricitabine and Abacavir Alone and in Combination with Additional Nucleoside Reverse Transcriptase Inhibitors. Antivir. Ther..

[141] Bonomini F., Rodella L.F., Rezzani R. (2015). Metabolic Syndrome, Aging and Involvement of Oxidative Stress. Aging Dis..

[142] Dos Santos W., Paes P., dos Santos A., Machado D., Navarro A., Frenandes A. (2013). Impact of progressive resistance training in Brazilian HIV patients with lipodystrophy. J. AIDS Clin. Res..

[143] Sharma B. (2011). Anti-HIV-1 drug toxicity and management strategies. Neurobehav. HIV Med..

[144] Csányi G. (2014). Oxidative Stress in Cardiovascular Disease.

[145] McColl D.J., Margot N., Chen S.-S., Harris J., Borroto-Esoda K., Miller M.D. (2011). Reduced Emergence of the M184V/I Resistance Mutation When Antiretroviral-Naïve Subjects Use Emtricitabine Versus Lamivudine in Regimens Composed of Two NRTIs Plus the NNRTI Efavirenz. HIV Clin. Trials.

[146] Hong S.Y., Jonas A., DeKlerk M., Shiningavamwe A., Desta T., Badi A., Morris L., Hunt G.M., Ledwaba J., Sheehan H.B. (2015). Population-Based Surveillance of HIV Drug Resistance Emerging on Treatment and Associated Factors at Sentinel Antiretroviral Therapy Sites in Namibia. JAIDS J. Acquir. Immune Defic. Syndr..

[147] Gulick R.M., Ribaudo H.J., Shikuma C.M., Lustgarten S., Squires K.E., Meyer W.A., Acosta E.P., Schackman B.R., Pilcher C.D., Murphy R.L. (2004). Triple-nucleoside regimens versus efavirenz-containing regimens for the initial treatment of HIV-1 infection. N. Engl. J. Med..

[148] Robbins G.K., De Gruttola V., Shafer R.W., Smeaton L.M., Snyder S.W., Pettinelli C., Dubé M.P., Fischl M.A., Pollard R.B., Delapenha R. (2003). Comparison of sequential three-drug regimens as initial therapy for HIV-1 infection. N. Engl. J. Med..

[149] Rakhmanina N.Y., la Porte C.J. (2012). Therapeutic drug monitoring of antiretroviral drugs in the management of human immunodeficiency virus infection. Ther. Drug Monit. Newer Drugs Biomark..

[150] EACS (2011). European AIDS Clinical Society Guidelines. Amsterdam, The Netherlands, Version 6. https://www.eacsociety.org/media/2011_eacsguidelines-v6.0-english_oct.pdf.

[151] Gilks C.F., Crowley S., Ekpini R., Gove S., Perriens J., Souteyrand Y., Sutherland D., Vitoria M., Guerma T., De Cock K. (2006). The WHO public-health approach to antiretroviral treatment against HIV in resource-limited settings. Lancet.

[152] Kahana S.Y., Rohan J., Allison S., Frazier T.W., Drotar D. (2012). A Meta-Analysis of Adherence to Antiretroviral Therapy and Virologic Responses in HIV-Infected Children, Adolescents, and Young Adults. AIDS Behav..

[153] Sliwa K., Hilfiker-Kleiner D., Petrie M.C., Mebazaa A., Pieske B., Buchmann E., Regitz-Zagrosek V., Schaufelberger M., Tavazzi L., van Veldhuisen D.J. (2010). Current state of knowledge on aetiology, diagnosis, management, and therapy of peripartum cardiomyopathy: A position statement from the Heart Failure Association of the European Society of Cardiology Working Group on peripartum cardiomyopathy. Eur. J. Hear. Fail..

[154] Schinazi R.F., Lloyd R.M., Nguyen M.H., Cannon D.L., McMillan A., Ilksoy N., Chu C.K., Liotta D.C., Bazmi H.Z., Mellors J.W. (1993). Characterization of human immunodeficiency viruses resistant to oxathiolane-cytosine nucleosides. Antimicrob. Agents Chemother..

[155] Maserati R., De Silvestri A., Uglietti A., Colao G., Di Biagio A., Bruzzone B., Di Pietro M., Re M.C., Tinelli C., Zazzi M. (2010). Emerging mutations at virological failure of HAART combinations containing tenofovir and lamivudine or emtricitabine. AIDS.

[156] Schinazi R.F., McMillan A., Cannon D., Mathis R., Lloyd R.M., Peck A., Sommadossi J.P., Clair M.S., Wilson J., A Furman P. (1992). Selective inhibition of human immunodeficiency viruses by racemates and enantiomers of cis-5-fluoro-1-[2-(hydroxymethyl)-1,3-oxathiolan-5-yl]cytosine. Antimicrob. Agents Chemother..

[157] Margot N.A., Enejosa J., Cheng A.K., Miller M.D., McColl D.J. (2009). Development of HIV-1 drug resistance through 144 weeks in antiretroviral-naive subjects on emtricitabine, tenofovir disoproxil fumarate, and efavirenz compared with lamivudine/zidovudine and efavirenz in study GS-01-934. JAIDS J. Acquir. Immune Defic. Syndr..

[158] Mugwanya K.K., John-Stewart G., Baeten J. (2017). Safety of oral tenofovir disoproxil fumarate-based HIV pre-exposure prophylaxis use in lactating HIV-uninfected women. Expert Opin. Drug Saf..

[159] Spinner C.D., Boesecke C., Zink A., Jessen H., Stellbrink H.J., Rockstroh J.K., Esser S. (2016). HIV pre-exposure prophylaxis (PrEP): A review of current knowledge of oral systemic HIV PrEP in humans. Infection.

[160] Fonner V.A., Dalglish S.L., Kennedy C.E., Baggaley R., O’Reilly K.R., Koechlin F.M., Rodolph M., Hodges-Mameletzis I., Grant R. (2016). Effectiveness and safety of oral HIV preexposure prophylaxis for all populations. AIDS.

[161] Borroto-Esoda K., E Vela J., Myrick F., Ray A.S., Miller M.D. (2005). In Vitro Evaluation of the Anti-HIV Activity and Metabolic Interactions of Tenofovir and Emtricitabine. Antivir. Ther..

[162] De Ruiter A., Taylor G.P., Clayden P., Dhar J., Gandhi K., Gilleece Y., Harding K., Hay P., Kennedy J., Low-Beer N. (2014). British HIV Association guidelines for the management of HIV infection in pregnant women 2012 (2014 interim review). HIV Med..

[163] Law W.P., Duncombe C.J., Mahanontharit A., Boyd M., Ruxrungtham K., Lange J.M.A., Phanuphak P., Cooper D.A., Dore G.J. (2004). Impact of viral hepatitis co-infection on response to antiretroviral therapy and HIV disease progression in the HIV-NAT cohort. AIDS.

[164] Zdanowicz M.M. (2006). The Pharmacology of HIV Drug Resistance. Am. J. Pharm. Educ..

[165] Hare C.B., Mellors J., Krambrink A., Su Z., Skiest D., Margolis D.M., Patel S.S., Barnas D., Frenkel L., Coombs R.W. (2008). Detection of nonnucleoside reverse-transcriptase inhibitor-resistant HIV-1 after discontinuation of virologically suppressive antiretroviral therapy. Clin. Infect. Dis..

[166] Carr A., Samaras K., Chisholm D.J., A Cooper D. (1998). Pathogenesis of HIV-1-protease inhibitor-associated peripheral lipodystrophy, hyperlipidaemia, and insulin resistance. Lancet.

[167] Del Valle L.G., Hernández R.G., Ávila J.P. (2013). Oxidative stress associated to disease progression and toxicity during antiretroviral therapy in human immunodeficiency virus infection. J. Virol. Microbiol..

[168] Awodele O., O Olayemi S., A Nwite J., A Adeyemo T. (2011). Investigation of the levels of oxidative stress parameters in HIV and HIV-TB co-infected patients. J. Infect. Dev. Ctries..

[169] Pinti M., Nasi M., Gibellini L., Roat E., De Biasi S., Bertoncelli L., Cossarizza A. (2010). The Role of Mitochondria in HIV Infection and Its Treatment. J. Exp. Clin. Med..

[170] Mandas A., Iorio E.L., Congiu M.G., Balestrieri C., Mereu A., Cau D., Dessì S., Curreli N. (2009). Oxidative Imbalance in HIV-1 Infected Patients Treated with Antiretroviral Therapy. J. Biomed. Biotechnol..

[171] Apostolova N., Gomez-Sucerquia L., Moran A., Alvarez A., Blas-Garcia A., Esplugues J. (2010). Enhanced oxidative stress and increased mitochondrial mass during Efavirenz-induced apoptosis in human hepatic cells. J. Cereb. Blood Flow Metab..

[172] Estrada V., Monge S., Garre G., Sobrino P., Masiá M., Berenguer J., Portilla J., Viladés C., Martinez E., Blanco J. (2016). Relationship between plasma bilirubin level and oxidative stress markers in HIV-infected patients on atazanavir-vs. efavirenz-based antiretroviral therapy. HIV Med..

[173] Elias A., Ijeoma O., Edikpo N.J., Oputiri D., Geoffrey O.-B.P. (2013). Tenofovir Renal Toxicity: Evaluation of Cohorts and Clinical Studies—Part One. Pharmacol. Pharm..

[174] Fernandez-Fernandez B., Montoya-Ferrer A., Sanz A.B., Sanchez-Niño M.D., Izquierdo M.C., Poveda J., Sainz-Prestel V., Ortiz-Martin N., Parra-Rodriguez A., Selgas R. (2011). Tenofovir Nephrotoxicity: 2011 Update. AIDS Res. Treat..

[175] Shafi T., Choi M., Racusen L., Spacek L., Berry C., Atta M., Fine D. (2011). Ritonavir-induced acute kidney injury: Kidney biopsy findings and review of literature. Clin. Nephrol..

[176] Boffito M. (2004). From concept to care: Pharmacokinetic boosting of protease inhibitors. PRN Noteb..

[177] Patel A.K., Ranjan R.R., Patel A.R., Patel J.K., Patel K.K. (2010). Tenofovir-associated renal dysfunction in clinical practice: An observational cohort from western India. Indian J. Sex. Transm. Dis. AIDS.

[178] Cao Y., Han Y., Xie J., Cui Q., Zhang L., Li Y., Li Y., Song X., Zhu T., Li T. (2013). Impact of a tenofovir disoproxil fumarate plus ritonavir-boosted protease inhibitor-based regimen on renal function in HIV-infected individuals: A prospective, multicenter study. BMC Infect. Dis..

[179] Grigsby I.F., Pham L., Mansky L.M., Gopalakrishnan R., Mansky K.C. (2010). Tenofovir-associated bone density loss. Ther. Clin. Risk Manag..

[180] Moss D.M., Neary M., Owen A. (2014). The role of drug transporters in the kidney: Lessons from tenofovir. Front. Pharmacol..

[181] Yeligar S.M., Guidot D.M., Brown L.A.S., Cribbs S.K. (2015). HIV Infection Induces NADPH Oxidase Expression in Alveolar Macrophages of Otherwise Healthy Subjects. C55 HIV-Associated Lung Diseases and Infections.

[182] Wang X., Liao D., Lin P.H., Yao Q., Chen C. (2009). Highly active antiretroviral therapy drugs inhibit in vitro cholesterol efflux from human macrophage-derived foam cells. Lab. Investig..

[183] So-Armah K., Freiberg M.S. (2018). HIV and Cardiovascular Disease: Update on Clinical Events, Special Populations, and Novel Biomarkers. Curr. HIV/AIDS Rep..

[184] Brouwer E.S., Napravnik S., Eron J.J., Stalzer B., Floris-Moore M., Simpson R.J., Stürmer T. (2014). Effects of Combination Antiretroviral Therapies on the Risk of Myocardial Infarction Among HIV Patients. Epidemiology.

[185] Marcel A.K., Ekali L.G., Eugene S., Arnold O.E., Sandrine E.D., Von der Weid D., Gbaguidi E., Ngogang J., Mbanya J.C. (2011). The effect of Spirulina platensis versus soybean on insulin resistance in HIV-infected patients: A randomized pilot study. Nutrients.

[186] Winter F.S., Emakam F., Kfutwah A., Hermann J., Azabji-Kenfack M., Krawinkel M.B. (2014). The Effect of Arthrospira platensis Capsules on CD4 T-Cells and Antioxidative Capacity in a Randomized Pilot Study of Adult Women Infected with Human Immunodeficiency Virus Not under HAART in Yaoundé, Cameroon. Nutrients.

[187] Ngo-Matip M.-E., Pieme C.A., Azabji-Kenfack M., Biapa P.C.N., Germaine N., Heike E., Moukette B.M., Emmanuel K., Philippe S., Mbofung C.M. (2014). Effects of Spirulina platensis supplementation on lipid profile in HIV–infected antiretroviral naïve patients in Yaounde-Cameroon: A randomized trial study. Lipids Health Dis..

[188] Ngo-Matip M.E., Pieme C.A., Azabji-Kenfack M., Moukette B.M., Korosky E., Stefanini P., Ngogang J.Y., Mbofung C.M. (2015). Impact of daily supplementation of Spirulina platensis on the immune system of naïve HIV-1 patients in Cameroon: A 12-months single blind, randomized, multicenter trial. Nutr. J..

[189] Koskey S., Okoth F., Waihenya R. (2013). Monitoring of CD4+ T-cell counts in HIV infected patients on Arthrospira platensis supplement in Kisumu, Kenya. Afr. J. Health Sci..

[190] Moor V.J.A., Pieme C.A., Nkeck J.R., Nya P.C.B., Mondinde G.I., Amazia F., Kouanfack C., Assoumou M.C.O., Ngogang J. (2021). Effects of Spirulina platensis on the Immune Status, Inflammatory and Oxidative Markers of HIV Patients on Antiretroviral Therapy in Cameroon. Acta Sci. Pharm. Sci..

[191] Antonelli M., Donelli D. (2022). Effects of Spirulina on CD4+ T-Lymphocyte Count in Patients with HIV Infection: A Literature Review. Biol. Life Sci. Forum.

